# Pleuropulmonary pathologies in the early phase of acute pancreatitis correlate with disease severity

**DOI:** 10.1371/journal.pone.0263739

**Published:** 2022-02-07

**Authors:** Ina Luiken, Stephan Eisenmann, Jakob Garbe, Hanna Sternby, Robert C. Verdonk, Alexandra Dimova, Povilas Ignatavicius, Lucas Ilzarbe, Peeter Koiva, Anne K. Penttilä, Sara Regnér, Johannes Dober, Walter A. Wohlgemuth, Richard Brill, Patrick Michl, Jonas Rosendahl, Marko Damm

**Affiliations:** 1 Department of Internal Medicine I, University Hospital Halle, Martin-Luther-University Halle-Wittenberg, Halle (Saale), Germany; 2 Department of Surgery, Institution of Clinical Sciences Malmö, Lund University, Malmö, Sweden; 3 Department of Gastroenterology, St. Antonius Ziekenhuis, Nieuwegein, The Netherlands; 4 Department of Surgery, University Hospital for Emergency Medicine “Pirogov”, Sofia, Bulgaria; 5 Department of Surgery, Lithuanian University of Health Sciences, Kaunas, Lithuania; 6 Department of Gastroenterology, Hospital del Mar, Barcelona, Spain; 7 Department of Gastroenterology, East Tallinn Central Hospital, Tallinn, Estonia; 8 Department of Surgery, Helsinki University Hospital and University of Helsinki, Helsinki, Finland; 9 Department of Radiology, University Hospital Halle, Martin-Luther-University Halle-Wittenberg, Halle (Saale), Germany; UKSH Campus Lübeck, GERMANY

## Abstract

**Background:**

Respiratory failure worsens the outcome of acute pancreatitis (AP) and underlying factors might be early detectable.

**Aims:**

To evaluate the prevalence and prognostic relevance of early pleuropulmonary pathologies and pre-existing chronic lung diseases (CLD) in AP patients.

**Methods:**

Multicentre retrospective cohort study. Caudal sections of the thorax derived from abdominal contrast enhanced computed tomography (CECT) performed in the early phase of AP were assessed. Independent predictors of severe AP were identified by binary logistic regression analysis. A one-year survival analysis using Kaplan-Meier curves and log rank test was performed.

**Results:**

358 patients were analysed, finding pleuropulmonary pathologies in 81%. CECTs were performed with a median of 2 days (IQR 1–3) after admission. Multivariable analysis identified moderate to severe or bilateral pleural effusions (PEs) (OR = 4.16, 95%CI 2.05–8.45, p<0.001) and pre-existing CLD (OR = 2.93, 95%CI 1.17–7.32, p = 0.022) as independent predictors of severe AP. Log rank test showed a significantly worse one-year survival in patients with bilateral compared to unilateral PEs in a subgroup.

**Conclusions:**

Increasing awareness of the prognostic impact of large and bilateral PEs and pre-existing CLD could facilitate the identification of patients at high risk for severe AP in the early phase and thus improve their prognosis.

## Introduction

Acute pancreatitis (AP) is one of the most frequent reasons for hospital admissions due to gastrointestinal diseases with increasing incidence and remains to be a major clinical and economic burden for health care systems in industrialised countries [1–3].

Three severity grades depending on the development and duration of organ failure and local or systemic complications are distinguished according to the revised Atlanta Classification. Particularly severe AP with infected pancreatic necrosis is associated with high mortality rates [4]. Furthermore, AP can be divided into an early and late phase. The early phase usually lasts for about one week and is characterised by systemic inflammatory response syndrome (SIRS), whereas the late phase is defined as persistent systemic inflammation or by local complications [4].

Organ failure can manifest in different systems, whereas respiratory failure is particularly common and associated with increased in-hospital mortality [5–7]. Previous studies suggested that chest pathologies such as pleural effusions and pulmonary infiltrations, detected radiographically in the first 24 hours of AP, might be associated with a necrotising course of AP and increased mortality risk [8–10]. Pleural effusions, detectable in chest radiography, are included in the BISAP- and PANC 3-Score, predicting in-hospital mortality or severity of AP, respectively [11, 12]. However, all existing scores show modest accuracy in predicting worse outcome [13]. As such, in order to improve the scoring systems, it will be necessary to identify new predictors or to specify the existing ones more accurately.

Due to the prognostic relevance of respiratory failure, this study aims to investigate the occurrence of early pleuropulmonary pathologies detected in contrast enhanced computed tomography (CECT) of patients with AP. Furthermore, these findings and pre-existing pulmonary comorbidities are evaluated as independent predictors for the development of severe AP to enable more accurate early identification of these patients in the future.

## Methods

### Data collection

In total, data of 395 patients hospitalised with AP in the period from January 2010 to December 2018 from seven European centres were screened for eligibility. Only patients with their first episode of AP, absent chronic pancreatitis and CECT imaging performed in the early phase of AP were included. Patients of this study have also partly been reported in two recent publications with distinct outcomes [14, 15]. After the screening process, 358 patients were included in the study (**S1 Table**), whereas 37 patients were excluded for the following reasons: underlying chronic pancreatitis or preceding episodes of AP (n = 17) or no CECT imaging in early phase of AP (n = 20). AP was diagnosed following guideline recommendations if at least two of the following three criteria were met: 1. acute abdominal pain, 2. increased serum amylase/ lipase level (elevated ≥3 times of the upper limit) or 3. characteristic morphological findings of AP in imaging [16].

The clinical data were collected from patient files at each centre, coded and transferred as fully anonymised data for analysis. The 1-year survival rate was only available for the centre of Halle, Germany. The ethics committee of the Martin-Luther-University Halle-Wittenberg provided ethical approval on the 17th of February 2021 (Number: 2021–037). The study was also approved previously by all local institutional review boards of participating centres (Kaunas Regional Biomedical Research Ethics Committee; Regional ethics committee at Lund University (2009/415); East Tallinn Central Hospital Research committee (1.1-19/48-12); Comitè Ètic d’Investigació Clínica (CEIC)—Parc de Salut MAR (2013/5069/I); Ethics Committee of the HUS Hospital district, Finland; Local ethics committee of the University Hospital for Emergency Medicine “Pirogov”, Bulgaria) [14, 15]. According to their decisions, gaining informed consent was not required due to the retrospective character of this study.

### Pleuropulmonary changes

**Fig 1:**
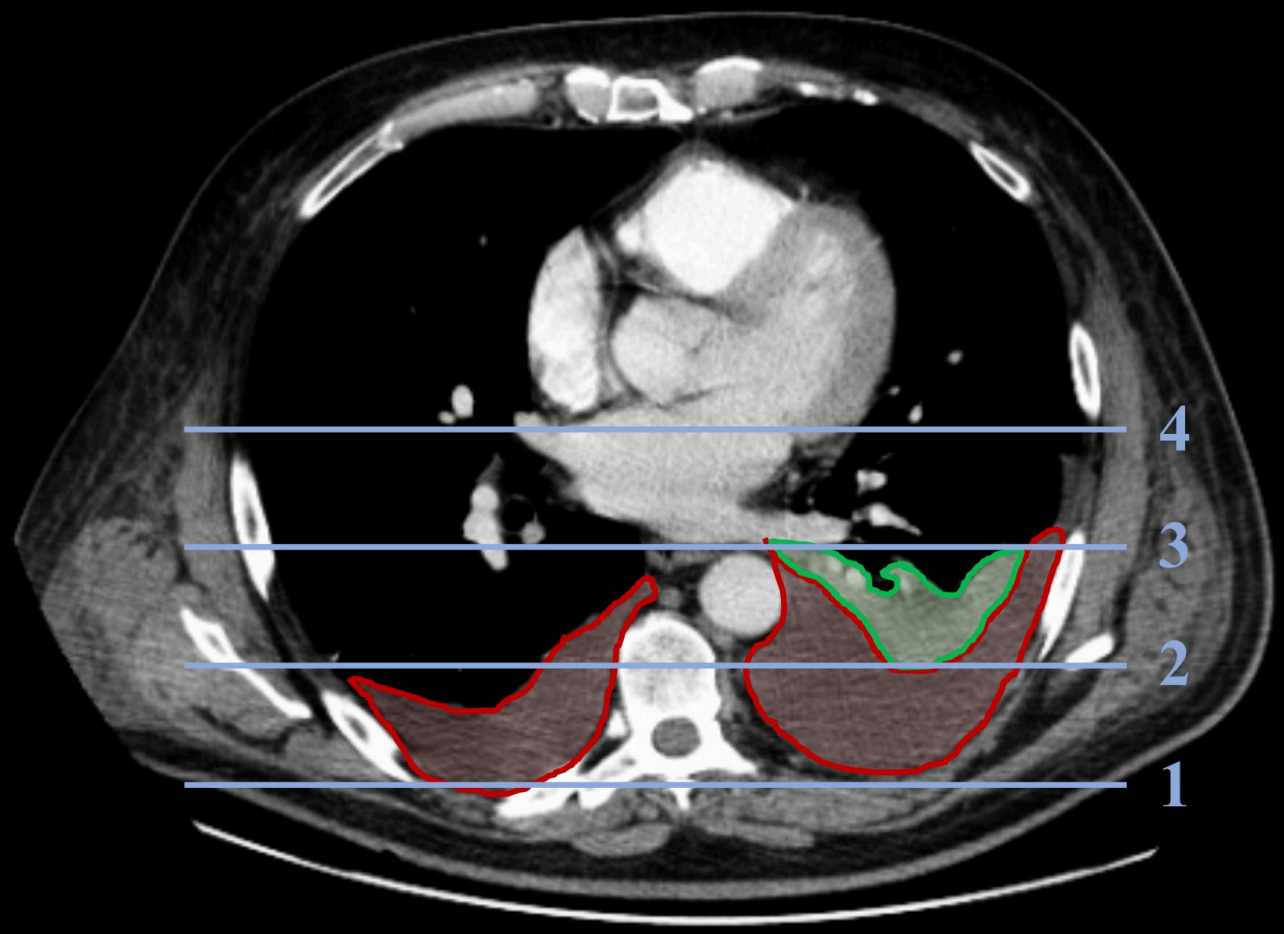
Exemplary representation of CECT imaging (mediastinal window) with unilateral dystelectasis (green) and severe, bilateral pleural effusions (red). Pleuropulmonary changes were analysed in the caudal sections of the thorax captured on the abdominal CECT (contrast enhanced computed tomography) scan. Thorax CECTs were additionally screened for pathologies, if available. The size of pleural effusions was determined in the transverse plane of the lung and mediastinal window. The area dorsal the midline of the thorax was divided into three parts similar in size (blue lines). Pleural effusions were classified as “low” if they remained dorsal line 2, as “moderate” if exceeding line 2, but not line 3 and as “severe” if exceeding line 3.

To evaluate pleuropulmonary changes, caudal sections of the thorax captured on CECT of the abdomen were analysed. Thorax CECTs were additionally screened for pathologies, when available. Images were analysed with the programmes PACS (Picture Archiving and Communication System, Dedalus Healthcare Systems Group, Florence, Italy) and InVesalius 3.1 (Centro de Tecnologia da Informação Renato Archer, Campinas, SP, Brazil). The CECT scans were reviewed (I.L.) and findings subsequently verified by an experienced radiologist (J.D.) and pulmonologist (S.E.) regarding the following findings: presence of pleural effusions (PEs) including localisation and amount, presence of dystelectases and/ or pleural contrast enhancement and elevation of the diaphragm on the left side. Elevation of the hemidiaphragm was analysed by comparing the height of the left and the right portion. **Fig 1** shows exemplary pleuropulmonary findings and illustrates how the amount of PE was determined.

For this purpose, the area dorsal to the midline of the thorax was divided into three parts with similar size. PE was classified as “low” if it remained dorsal line 2, “moderate” if it was exceeding line 2, but not line 3 and “severe” if it was exceeding line 3. This pleural fluid quantification method was adopted from the study of Lo Gullo et al., in which grading of pleural fluid on postmortem transverse CT images correlated significantly with the volume of pleural fluids on autopsy [17].

### Statistical analysis

The collected data was analysed using SPSS statistics software version 27 (IBM Inc., Armonk, NY, USA). Categorical variables were reported as frequency, percentages and continuous variables as median with interquartile range (IQR Q1 –Q3). Independent samples were compared applying the Mann-Whitney U or Pearson’s chi-square test, as appropriate. To identify independent predictors of severe AP, potential variables with significant associations were identified in univariable analysis. A two-sided p-value <0.05 was considered statistically significant. All variables showing a p-value <0.05 in univariable analysis were included in a multivariable regression model, in which variables were selected by forward elimination. In addition, to compute adjusted odds ratios, all variables significant in univariable analysis were included in a regression model together with age and gender using the full model approach. Because several variables describing PEs were significantly associated, the most significant variable (i.e. combination of localisation and amount) was included. Survival analysis was performed using Kaplan-Meier curves and log-rank test.

## Results

### Patient characteristics

In total, 358 patients from seven European centres were enrolled in this study. The baseline characteristics are shown in **Table 1**. A majority of the patients (165/358, 46.1%) developed a moderately severe AP, whereas 41.1% (147/358) had a mild and 12.8% (46/358) a severe disease course according to the revised Atlanta Classification [4]. **S1 Table** summarises the different study centres with their respective proportion of recruited patients. AP was most commonly caused by biliary obstruction (132/358, 36.9%), followed by alcohol consumption (115/358, 32.1%). A pre-existing chronic lung disease (CLD), such as chronic obstructive lung disease (COPD), asthma, interstitial or emphysematous lung pathologies was documented in 31 cases (31/358, 8.7%).

**Table 1:** Baseline characteristics of the study population (n = 358).

		Severity of acute pancreatitis (n = 358) ^†^		
	Total	Mild	Moderately severe	Severe
	n = 358 (100.0%)	n = 147 (41.1%)	n = 165 (46.1%)	n = 46 (12.8%)
**Gender** (%)				
• Female	146 (40.8)	56 (38.1)	71 (43.0)	19 (41.3)
• Male	212 (59.2)	91 (61.9)	94 (57.0)	27 (58.7)
**Age** (years)*	58 (45–72)	62 (48–73)	55 (42–67)	62 (47–76)
**Aetiology** (%)				
• Biliary	132 (36.9)	60 (40.8)	60 (36.4)	12 (26.1)
• Alcohol	115 (32.1)	40 (27.2)	62 (37.6)	13 (28.3)
• Idiopathic	75 (20.9)	31 (21.1)	34 (20.6)	10 (21.7)
• Others**	36 (10.1)	16 (10.9)	9 (5.5)	11 (23.9)
**BMI** (kg/m^2^)*	28.0 (24.8–31.3)^1^	27.7 (24.7–30.8)^2^	28.6 (25.6–32.0)^3^	27.5 (24.5–31.1)^4^
**CLD** (%)				
• No	324 (90.5)	134 (91.2)	154 (93.3)	36 (78.3)
• Yes	31 (8.7)^5^	12 (8.2)^6^	11 (6.7)	8 (17.4)^7^
**Ventilation** (%)				
• No	323 (90.2)	146 (99.3)^9^	161 (97.6)	16 (34.8)
• Yes	34 (9.5)^8^	-	4 (2.4)	30 (65.2)
**Organ failure** (%)				
• No	274 (76.5)	147 (100.0)	127 (77.0)	-
• Yes	84 (23.5)	-	38 (23.0)	46 (100.0)
• Transient	38 (10.6)	-	38 (23.0)	-
• Persistent	46 (12.8)	-	-	46 (100.0)
**In-hospital mortality** (%)				
• No	338 (94.4)	146 (99.3)	162 (98.2)	30 (65.2)
• Yes	20 (5.6)	1 (0.7)	3 (1.8)	16 (34.8)

Abbreviations: BMI = Body Mass Index, CLD = Chronic Lung Disease (chronic obstructive pulmonary disease, asthma, fibrosis or emphysema)

† According to the revised Atlanta classification

* Median (IQR)

** Others: Post-ERCP pancreatitis, autoimmune pancreatitis, pancreatitis due to lipid metabolic disorders or medication

Missings:

^1^ 60 (16.8%)

^2^ 30 (20.4%)

^3^ 27 (16.4%)

^4^ 3 (6.5%)

^5^ 3 (0.8%)

^6^ 1 (0.7%)

^7^ 2 (4.3%)

^8^ 1 (0.3%)

^9^ 1 (0.7%)

As complication of AP, 23.5% (84/358) of the patients showed cardiovascular, respiratory or renal failure, of which 54.8% (46/84) had persistent (>48h) and 45.2% (38/84) transient (<48h) organ failure. 34 (9.5%) patients required artificial (invasive and non-invasive) ventilation. The overall in-hospital mortality was 5.6% (20/358). Patients with severe AP had a higher in-hospital mortality (16/46, 34.8%) than patients with mild (1/147, 0.7%) or moderately severe AP (3/165, 1.8%).

### Early pleuropulmonary findings in acute pancreatitis

**Fig 2:**
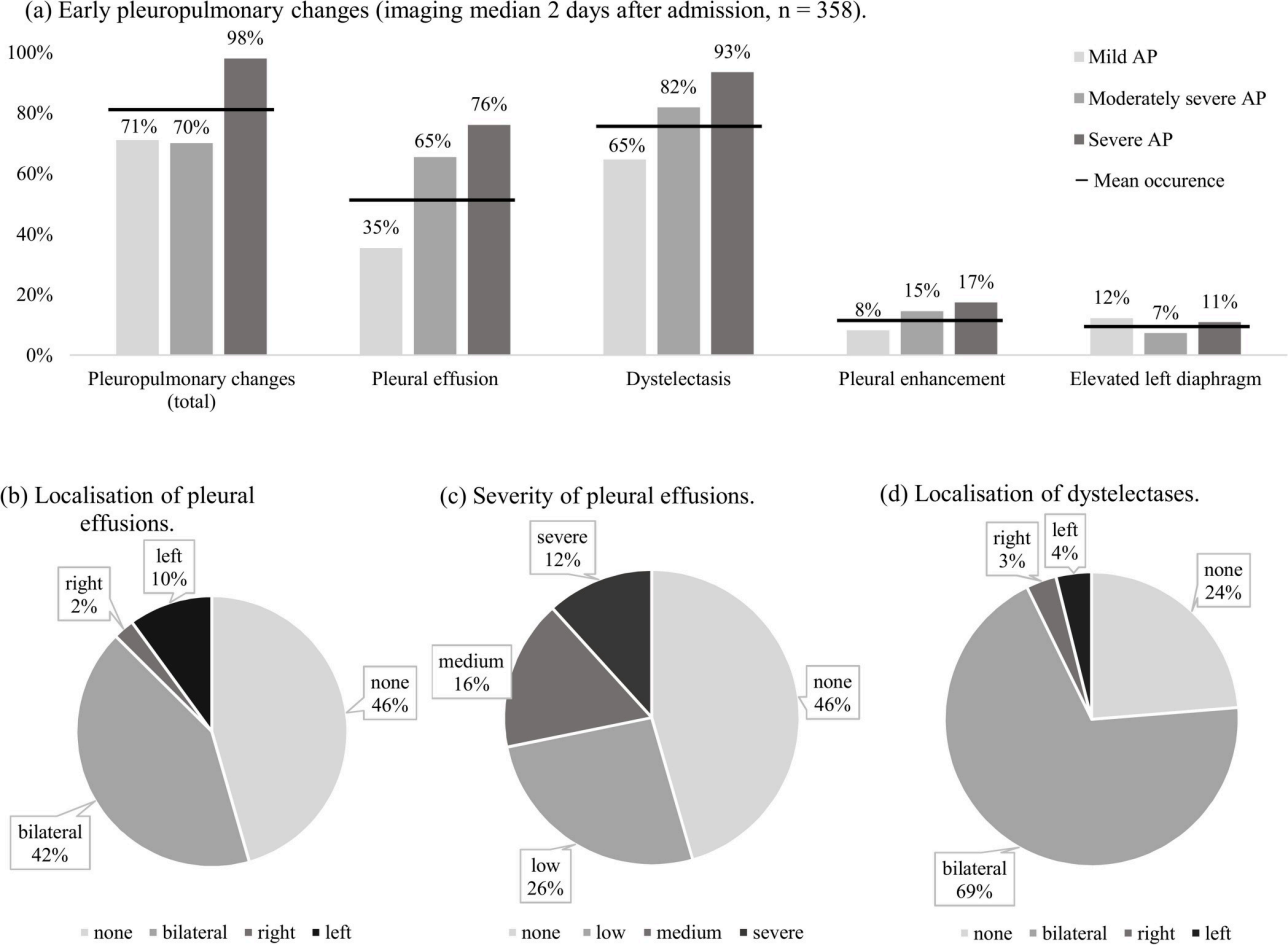
Distribution of early pleuropulmonary findings in CECT imaging from patients with acute pancreatitis (n = 358). Proportions of all pleuropulmonary findings in the study population with subdivisions into the different grades of acute pancreatitis (AP) are depicted in (a). The median timing of CECT imaging was 2 days after admission. Graphs (b) and (c) show the detailed localisation and severity of pleural effusions (PEs) and (d) the distribution of dystelectases. In total, 81% (289/358) of patients had pleuropulmonary changes in CECT imaging and the occurrence mostly increased with severity of AP, e.g. approximately one-third of patients with mild AP showed PEs (52/147, 35%), whereas PEs appeared in 76% (35/46) in patients with severe AP. In most cases (273/358, 76%) the detected findings were dystelectases. More than 90% (247/273, 91%) of dystelectases appeared bilateral. Pleural enhancement was found in 12% (44/358) and an elevation of the left diaphragm in 10% (35/358). More than half of the patients had PE (195/358, 54%), that was mostly (150/195, 77%) bilateral. If unilateral, PE was observed particularly in the left pleural cavity (36/45, 80%). When PE was prevalent, its amount was classified as low, moderate or severe in 48% (94/195), 30% (59/195) and 22% (42/195) of the cases, respectively.

The median time between CECT imaging and admission was 2 days (IQR 1–3), with no significant differences between the severity groups (**Table 2**). In total, 81% (289/358) of all patients showed pleuropulmonary changes in CECT imaging (**Fig 2**). 76.3% of the patients showed dystelectases (273/358, 76.3%), from which 90.5% (247/273) were bilateral. More than half of the patients showed PEs (195/358, 54.5%). PEs also appeared mostly bilateral (150/195, 76.9%). If unilateral (45/195, 23.1%), PEs occurred particularly in the left pleural cavity (36/45, 80%). If PEs were prevalent, the amount was classified as low, moderate or severe in 48.2% (94/195), 30.3% (59/195) and 21.5% (42/195) of the cases, respectively. Pleural enhancement and an elevated left diaphragm was found in 12% (44/358) and 10% (35/358) of the patients, respectively. Overall, the occurrence of pleuropulmonary changes increased with the severity of AP. For example, PEs were found in 35%, 65% and 76% in mild, moderately severe and severe AP, respectively.

**Table 2:** Early pleuropulmonary findings in patients with acute pancreatitis (n = 358).

		Severity of acute pancreatitis (n = 358) ^†^		
	Total	Mild	Moderately severe	Severe
	n = 358 (100.0%)	n = 147 (41.1%)	n = 165 (46.1%)	n = 46 (12.8%)
**Timing of CECT**				
**after admission** (days)*	2 (1–3)	2 (1–3)	2 (1–4)	2 (1–3)
**Pleural effusion** (%)				
• None	163 (45.5)	95 (64.6)	57 (34.5)	23.9)
• Total	195 (54.5)	52 (35.4)	108 (65.5)	76.1)
• Bilateral	150 (41.9)	31 (21.1)	88 (53.3)	67.4)
• Unilateral	45 (12.6)	21 (14.3)	20 (12.1)	(8.7)
• Right	9 (2.5)	6 (4.1)	3 (1.8)	(0.0)
• Left	36 (10.1)	15 (10.2)	17 (10.3)	4 (8.7)
**Amount of pleural effusion** (%)				
• None	163 (45.5)	95 (64.6)	57 (34.5)	23.9)
• Low	94 (26.3)	39 (26.5)	43 (26.1)	26.1)
• Moderate	59 (16.5)	6 (4.1)	41 (24.8)	26.1)
• Severe	42 (11.7)	7 (4.8)	24 (14.5)	11 (23.9)
**Dystelectasis** (%)				
• None	85 (23.7)	52 (35.4)	30 (18.2)	(6.5)
• Total	273 (76.3)	95 (64.6)	135 (81.8)	93.5)
• Bilateral	247 (69.0)	76 (51.7)	129 (78.2)	91.3)
• Unilateral	26 (7.3)	19 (12.9)	6 (3.6)	(2.2)
• Right	12 (3.4)	9 (6.1)	3 (1.8)	(0.0)
• Left	14 (3.9)	10 (6.8)	3 (1.8)	1 (2.2)
**Pleural enhancement** (%)				
• None	314 (87.7)	135 (91.8)	141 (85.5)	(82.6)
• Total	44 (12.3)	12 (8.2)	24 (14.5)	(17.4)
• Bilateral	32 (8.9)	6 (4.1)	19 (11.5)	(15.2)
• Unilateral	12 (3.4)	6 (4.1)	5 (3.0)	(2.2)
• Right	4 (1.1)	1 (0.7)	3 (1.8)	(0.0)
• Left	8 (2.2)	5 (3.4)	2 (1.2)	1 (2.2)
**Elevated left diaphragm** (%)				
• No	323 (90.2)	129 (87.8)	153 (92.7)	89.1)
• Yes	35 (9.8)	18 (12.2)	12 (7.3)	5 (10.9)

Abbreviations: CECT = Contrast Enhanced Computed Tomography

† According to the revised Atlanta classification

* Median (IQR)

### Pleuropulmonary predictors of severe acute pancreatitis

To identify possible predictors of severe AP, the study population was divided into patients with mild and moderately severe (n = 312) versus patients with severe AP (n = 46) (**S2 Table**) [4]. There were no significant differences in age (57 vs. 62 years (median), p = 0.187), gender (female: 40.7% vs. 41.3%, p = 1.0) or BMI (28.1 vs. 27.5 (median), p = 0.36) between those groups. Univariable analysis showed that the presence of PEs itself, a moderate or severe amount, a bilateral localisation of PEs or a combination variable (presence of bilateral PEs or moderate to severe amount of PEs) were associated with severe AP (**S2 Table**). Moreover, the presence of bilateral dystelectases (p<0.001) or a known chronic lung disease (CLD, p = 0.036) also showed an association with severe AP. Next, the multivariable analysis was performed with two different approaches. All significant variables were included in a forward elimination model which showed that known CLD (OR = 2.927, 95% CI 1.171–7.319, p = 0.022) and moderate to severe or bilateral PEs (OR = 4.163, 95% CI 2.052–8.448, p<0.001) were independent predictors for severe AP (**Table 3**). Additionally, a full model approach was used to obtain age and gender adjusted odds ratios (aOR). Here, known CLD (aOR = 2.582, 95% CI = 1.014–6.578, p = 0.047) and moderate to severe or bilateral PEs (aOR = 3.027, 95% CI = 1.405–6.521, p = 0.005) were again identified as independent predictors for severe AP.

**Table 3:** Predictors of severe acute pancreatitis: Multivariable analysis (n = 358).

	Severe AP ^†^ (full model approach)			Severe AP ^†^ (forward elimination)		
	aOR	*p*-value	*95% CI*	*OR*	*p-value*	*95% CI*
**Gender**						
• Female	1 (ref)					
• Male	1.005	0.990	0.504–2.002	-	-	-
**Age**						
• <60 years	1 (ref)					
• ≥60 years	1.045	0.898	0.533–2.051	-	-	-
**Known CLD**						
• No	1 (ref)			(ref)		
• Yes	**2.582**	**0.047**	1.014–6.578	**2.927**	**0.022**	1.171–7.319
**Dystelectasis**						
• None/unilateral	1 (ref)					
• Bilateral	2.673	0.092	0.852–8.392	-	-	-
**Pleural effusion localisation and amount** (%)						
• None/mild/Unilateral	1 (ref)			(ref)		
• Severe/moderate/bilateral	**3.027**	**0.005**	1.405–6.521	**4.163**	**<0.001**	2.052–8.448

Abbreviations: CLD = Chronic Lung Disease (chronic obstructive pulmonary disease, asthma, fibrosis or emphysema), CI = Confidence Interval, OR = Odds Ratio, aOR = adjusted Odds Ratio, ref = reference

† According to the revised Atlanta classification; Severity was dichotomised into severe versus mild and moderately severe acute pancreatitis.

### One-year survival

Survival data was only available for the subpopulation from the centre of Halle, Germany (n = 90). During the one-year follow up 16 patients died (16/90, 17.8%). The aetiology was biliary obstruction in four patients, alcohol consumption in three, idiopathic in four and other causes (e.g. post-ERCP, autoimmune, medication) in five patients. Of the 16 patients, severity according to the revised Atlanta classification was classified as severe in ten and as moderately severe or mild in three patients, respectively. Overall, the median time to death after admission was 104 days (IQR 11–174). Survival data represent the all-cause mortality, information about the specific cause of death was not available.

**Fig 3:**
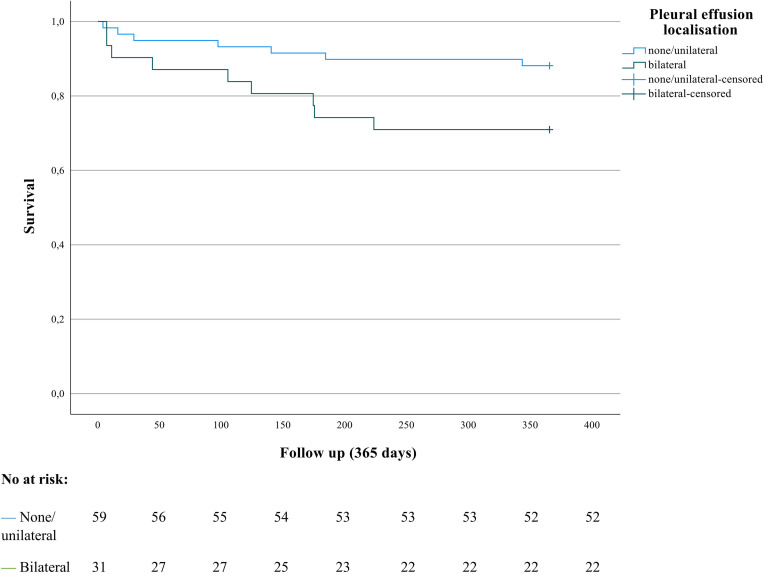
Bilateral pleural effusions in the early phase of acute pancreatitis are associated with worse one-year survival. Kaplan–Meier curves of patients with none or unilateral pleural effusions (PEs) (n = 59) versus patients with bilateral PEs (n = 31) in the early phase (median 2 days after admission) of acute pancreatitis (AP) are shown. Survival data was only available for a subgroup of patients (study centre Halle, Germany, n = 90). Hospitalised patients with AP were followed up for one year after admission. Overall, 16 patients (18%) died during follow up. Of patients with none/ unilateral PEs 12% (7/59) died, whereas 29% (9/31) of the patients with bilateral PEs died. Log rank test showed significant worse one-year survival in the group of patients with bilateral PEs compared to none/ unilateral PEs (p = 0.04).

Kaplan–Meier analysis and log rank test were performed for patients with none or unilateral PEs (n = 59) versus patients with bilateral PEs (n = 31, **Fig 3**). While there was no difference in 30d mortality (p = 0.4), log rank test showed a significant worse one-year survival in patients with bilateral PEs. 11.9% (7/59) of patients with none/ unilateral PE and 29% (9/31) with bilateral PEs died during 12 months of follow up (p = 0.04).

Moreover, independent predictors of severe AP identified in multivariable analysis demonstrated a tendency of worse one-year survival in log rank test (“known CLD” vs. “no CLD”, p = 0.07 and the subgroup of “moderate to severe or bilateral PEs” vs. “none, mild or unilateral PEs”, p = 0.07).

Compared to the patients for whom survival data was not available (n = 268), there were no significant differences in age, BMI and the severity of the pancreatitis. However, the subgroup from Halle contained significantly more male patients (64/90 (71%) vs. 148/268 (55%), p = 0.008), the aetiology of the pancreatitis was less frequently alcohol (20/90 (22%) vs. 95/268 (35%), p = 0.02) and there were more CLD prevalent (15/90 (17%) vs. 16/268 (6%), p = 0,002).

## Discussion

To predict the outcome in AP there is still a deficiency in reliable parameters or scores. As such, this international, multicentre, retrospective study with a large set of patients aimed to investigate pleuropulmonary pathologies on CECT in the early phase of AP, which might be used for severity prediction in the future. In our cohort, pleuropulmonary pathologies were found frequently and in particular bilateral and larger PEs correlated with a more severe course of the disease.

Overall, pleuropulmonary changes were detected in more than 80% of our patients and thus more often compared to previous reports, where the prevalence ranged from 14 to 48% [7, 8, 18–21]. Most of the former studies, however, analysed chest X-rays instead of CECTs, which are less sensitive in detecting pleuropulmonary pathologies [22]. Furthermore, the presence and amount of PEs in the early phase of AP increases over time and consequently measurements at earlier time points in former studies might also explain the divergent results [23]. Lastly, a different proportion of patients with severe AP in the reports with lower prevalence of pleuropulmonary findings could also explain discordant data, as we and others showed that these findings are more common in the severe disease course [10]. A recent single centre study of 309 consecutive AP patients analysed CECTs performed within the first two days after symptom onset and reported an occurrence of PEs and pulmonary consolidations in 40% and 48%, respectively, a finding closer to the prevalence rates observed in our cohort [18]. Consistent with our findings, increased PE volume and pulmonary consolidation were significantly associated with occurrence of severe AP and organ failure, but no multivariable analysis was performed in this study.

As such, our data showed for the first time that bilateral and/ or moderate to severe amounts of PEs, detected in the early phase of AP, are independent predictors of severe AP, whereas other observations like pleural enhancement or an elevated left hemidiaphragm are not.

The presence of PEs was identified as a predictor in former works and this variable was included in prognostic tools such as the Panc3 and the BISAP score [12, 24]. Of note, for the Panc3 score, the presence of PEs in the retrospective validation cohort of 238 patients was determined via chest X-ray, whereas the BISAP index was based on a large population-based database comprising >30.000 patients with AP, where PEs from both chest X-ray or CECT were noted. Although the correlation of PEs with severity in AP may be strong in general, a prospective comparison of existing prognostic scores revealed modest accuracy in predicting persistent organ failure at admission with areas under the curve ranging from 0.57 to 0.72 [13]. Here, our findings suggest that PEs should be differentiated with regard to their prognostic significance, as the presence of PEs in itself was no independent risk factor of severe AP. Thus, small and unilateral PEs can probably be neglected and should not be included in prognostic scores and a more specific inclusion of “predictive” PEs (i.e. bilateral and larger PEs) might improve prognostic accuracy. In line with our results, Peng and colleagues have shown, that the accuracy of the PE volume alone in predicting severe AP was comparable to the APACHE II and BISAP scores [18].

Our results also reveal that a known chronic lung disease (e.g. chronic obstructive pulmonary disease or asthma) is an independent predictor of severe AP in multivariable analysis. Except for APACHE II, this variable is not included in any other prognostic score used for AP patients, and has not been reported often yet. Recently, He and co-authors investigated risk factors of moderately severe and severe AP in elderly patients (≥60 years) and identified pre-existing pulmonary disease (OR 7.1) besides known predictors such as increased haematocrit level (OR 3.7) or PE (OR 5.0) to correlate with severity [25]. Because respiratory insufficiency is the most common organ failure in AP, it seems to be particularly crucial for the prognosis [5, 7, 25, 26].

Our observations are strengthened by another relevant endpoint, as we demonstrate in a subpopulation of 90 patients that bilateral PEs were significantly associated with impaired one-year survival in log rank test (p = 0.04). Noteworthy, there has also been a tendency for the subgroup of moderate to severe or bilateral PEs (p = 0.07) and CLD (p = 0.07) that however did not reach statistical significance most likely due to the small sample size investigated. Similarly, Dombernowsky et al. have shown, that AP patients with respiratory failure had an increased 30-day mortality [7]. Independent predictors of respiratory failure were age and a history of smoking, whereas an association with chronic obstructive lung disease was found in univariable, but not in multivariable analysis in this study.

The underlying pathophysiology for respiratory impairment in AP might be systemic inflammation that leads to a release of vasoactive and pro-inflammatory substances promoting an increasing permeability of the lung barrier with leakage of fluid into the alveolar spaces [7]. This respiratory impairment might additionally be exacerbated by pleuropulmonary pathologies, such as larger or bilateral PEs, with respiratory failure as consequence leading to a more severe individual course of the disease. Otherwise, the development of PEs may be related to an increased capillary permeability of the pleura induced by inflammatory processes in close proximity to the diaphragm, so that proteolytic enzymes from the pancreatic secretion may even harm the lung directly [21, 27]. Here, pancreaticopleural fistula may also cause severe effusions in AP patients [28]. Although, several concepts for the development of pleuropulmonary pathologies in AP have been postulated, further research is needed to increase understanding of the underlying mechanisms.

Our study comes along with several limitations that are to a large extent due to its retrospective nature. First, there might be a substantial selection bias, as only hospitalised patients with a CECT in the early phase of the disease were included. This selection might result in overrepresentation of severe AP cases in our study population and thereby overrate numbers of pleuropulmonary findings. Next, cranial sections of the abdominal CECT were analysed in most cases and consequently pleuropulmonary pathologies in the apical parts of the lungs were not recorded. As no imaging was performed before the episode of AP, it is elusive whether pleuropulmonary pathologies were newly acquired or pre-existing. Due to the retrospective data acquisition from patient files, probably not all previously known chronic lung diseases have been included. Furthermore, only the date of hospital admission was recorded and therefore the timing of the CECT related to symptom onset could not be specified. Finally, regarding survival analysis, as the data was only available for a subgroup of patients the results might not be representative for the entire cohort.

One of the relevant findings reported in our cohort is, that in contrast to unilateral and small PEs, bilateral and moderate to severe PEs are independent predictors of severe AP. Additionally, patients with a known CLD are at increased risk and as such need close surveillance. The implementation of these variables in prognostic scores could improve their predictive accuracy, which should be tested in prospective studies. For the diagnosis and classification of PEs, the abdominal CECT seems practical, however, ultrasound is more readily available, safe, fast, and almost equivalent in terms of diagnostic accuracy [22]. As a consequence, one might suggest assessing PEs by ultrasound in the early phase of AP routinely to substantiate the risk of a severe disease course. In addition, there may be therapeutic implications to improve the outcome: A consequent screening and drainage of larger PEs may reduce the rate of respiratory failure, however robust data in this regard is lacking and could be addressed by further investigations.

In summary, larger and bilateral PEs, detected in the early phase of AP and pre-existing CLD are independent predictors of a severe disease course. In addition, patients with bilateral PEs show a significant worse one-year survival. The proposed impact of these pathologies on the severity of AP seems substantial, but needs to be investigated in larger prospective studies, that also evaluate the prognostic capacity of the correlations.

## Supporting information

S1 Table Overview of study centres and included patients. (DOCX)

S2 Table Predictors of severe acute pancreatitis: Univariable analysis. (DOCX)

## References

[1] Roberts SE, Morrison-Rees S, John A, Williams JG, Brown TH, Samuel DG. The incidence and aetiology of acute pancreatitis across Europe. Pancreatology. 2017; 17:155–65. doi: 10.1016/j.pan.2017.01.005 . 28159463

[2] Forsmark CE, Vege SS, Wilcox CM. Acute Pancreatitis. N Engl J Med. 2016; 375:1972–81. doi: 10.1056/NEJMra1505202 . 27959604 PMC13220086

[3] Afghani E, Pandol SJ, Shimosegawa T, Sutton R, Wu BU, Vege SS, et al. Acute Pancreatitis-Progress and Challenges: A Report on an International Symposium. Pancreas. 2015; 44:1195–210. doi: 10.1097/MPA.0000000000000500 . 26465949 PMC4890478

[4] Banks PA, Bollen TL, Dervenis C, Gooszen HG, Johnson CD, Sarr MG, et al. Classification of acute pancreatitis—2012: revision of the Atlanta classification and definitions by international consensus. Gut. 2013; 62:102–11. doi: 10.1136/gutjnl-2012-302779 . 23100216

[5] Mofidi R, Duff MD, Wigmore SJ, Madhavan KK, Garden OJ, Parks RW. Association between early systemic inflammatory response, severity of multiorgan dysfunction and death in acute pancreatitis. The British journal of surgery. 2006; 93:738–44. doi: 10.1002/bjs.5290 . 16671062

[6] Garg PK, Madan K, Pande GK, Khanna S, Sathyanarayan G, Bohidar NP, et al. Association of extent and infection of pancreatic necrosis with organ failure and death in acute necrotizing pancreatitis. Clinical Gastroenterology and Hepatology. 2005; 3:159–66. doi: 10.1016/s1542-3565(04)00665-2 . 15704050

[7] Dombernowsky T, Kristensen MO, Rysgaard S, Gluud LL, Novovic S. Risk factors for and impact of respiratory failure on mortality in the early phase of acute pancreatitis. Pancreatology. 2016; 16:756–60. doi: 10.1016/j.pan.2016.06.664 . 27424478

[8] Talamini G, Uomo G, Pezzilli R, Billi P, Bassi C, Cavallini G, et al. Serum creatinine and chest radiographs in the early assessment of acute pancreatitis. American journal of surgery. 1999; 177:7–14. doi: 10.1016/s0002-9610(98)00296-7 . 10037300

[9] Lankisch PG, Dröge M, Becher R. Pulmonary infiltrations. Sign of severe acute pancreatitis. Int J Pancreatol. 1996; 19:113–5. doi: 10.1007/BF02805224 . 8723553

[10] Heller SJ, Noordhoek E, Tenner SM, Ramagopal V, Abramowitz M, Hughes M, et al. Pleural effusion as a predictor of severity in acute pancreatitis. Pancreas. 1997; 15:222–5. doi: 10.1097/00006676-199710000-00002 . 9336784

[11] Papachristou GI, Muddana V, Yadav D, O’Connell M, Sanders MK, Slivka A, et al. Comparison of BISAP, Ranson’s, APACHE-II, and CTSI scores in predicting organ failure, complications, and mortality in acute pancreatitis. Am J Gastroenterol. 2010; 105:435–41; quiz 442. doi: 10.1038/ajg.2009.622 . 19861954

[12] Wu BU, Johannes RS, Sun X, Tabak Y, Conwell DL, Banks PA. The early prediction of mortality in acute pancreatitis: a large population-based study. Gut. 2008; 57:1698–703. doi: 10.1136/gut.2008.152702 . 18519429

[13] Mounzer R, Langmead CJ, Wu BU, Evans AC, Bishehsari F, Muddana V, et al. Comparison of existing clinical scoring systems to predict persistent organ failure in patients with acute pancreatitis. Gastroenterology. 2012; 142:1476–82. doi: 10.1053/j.gastro.2012.03.005 . 22425589

[14] Sternby H, Mahle M, Linder N, Erichson-Kirst L, Verdonk RC, Dimova A, et al. Mean muscle attenuation correlates with severe acute pancreatitis unlike visceral adipose tissue and subcutaneous adipose tissue. United European Gastroenterol J. 2019; 7:1312–20. doi: 10.1177/2050640619882520 . 31839956 PMC6893994

[15] Sternby H, Verdonk RC, Aguilar G, Dimova A, Ignatavicius P, Ilzarbe L, et al. Significant inter-observer variation in the diagnosis of extrapancreatic necrosis and type of pancreatic collections in acute pancreatitis—An international multicenter evaluation of the revised Atlanta classification. Pancreatology. 2016; 16:791–7. doi: 10.1016/j.pan.2016.08.007 . 27592205

[16] Working Group IAP/APA Acute Pancreatitis Guidelines. IAP/APA evidence-based guidelines for the management of acute pancreatitis. Pancreatology. 2013; 13:e1–15. doi: 10.1016/j.pan.2013.07.063 . 24054878

[17] Lo Gullo R, Mishra S, Lira DA, Padole A, Otrakji A, Khawaja RDA, et al. Quantification of interstitial fluid on whole body CT: comparison with whole body autopsy. Forensic Sci Med Pathol. 2015; 11:488–96. Epub 2015/11/05. doi: 10.1007/s12024-015-9728-y . 26541472

[18] Peng R, Zhang L, Zhang Z-M, Wang Z-Q, Liu G-Y, Zhang X-M. Chest computed tomography semi-quantitative pleural effusion and pulmonary consolidation are early predictors of acute pancreatitis severity. Quant Imaging Med Surg. 2020; 10:451–63. doi: 10.21037/qims.2019.12.14 . 32190570 PMC7063295

[19] Rathnakar SK, Vishnu VH, Muniyappa S, Prasath A. Accuracy and Predictability of PANC-3 Scoring System over APACHE II in Acute Pancreatitis: A Prospective Study. J Clin Diagn Res. 2017; 11:PC10–PC13. doi: 10.7860/JCDR/2017/23168.9375 . 28384928 PMC5376859

[20] Lankisch PG, Dröge M, Becher R. Pleural effusions: a new negative prognostic parameter for acute pancreatitis. Am J Gastroenterol. 1994; 89:1849–51. 7942681

[21] Chelliah T, Werge M, Merc A-I, Bisgaard T, Hansen EF, Hansen EF, et al. Pulmonary dysfunction due to combination of extra-pulmonary causes and alveolar damage is present from first the day of hospital admission in the early phase of acute pancreatitis. Pancreatology. 2019; 19:519–23. doi: 10.1016/j.pan.2019.04.009 . 31036490

[22] Ashton-Cleary DT. Is thoracic ultrasound a viable alternative to conventional imaging in the critical care setting. Br J Anaesth. 2013; 111:152–60. Epub 2013/04/12. doi: 10.1093/bja/aet076 . 23585400

[23] Huang H, Chen W, Tang G, Liang Z, Qin M, Qin M, et al. Optimal timing of contrast-enhanced computed tomography in an evaluation of severe acute pancreatitis-associated complications. Experimental and therapeutic medicine. 2019; 18:1029–38. doi: 10.3892/etm.2019.7700 . 31363364 PMC6614731

[24] Brown A, James-Stevenson T, Dyson T, Grunkenmeier D. The panc 3 score: a rapid and accurate test for predicting severity on presentation in acute pancreatitis. J Clin Gastroenterol. 2007; 41:855–8. doi: 10.1097/01.mcg.0000248005.73075.e4 . 17881932

[25] He F, Zhu H-M, Li B-Y, Li X-C, Yang S, Wang Z, et al. Factors predicting the severity of acute pancreatitis in elderly patients. Aging clinical and experimental research. (2021); 33:183–92. doi: 10.1007/s40520-020-01523-1 . 32185694

[26] Wig JD, Bharathy KGS, Kochhar R, Yadav TD, Kudari AK, Doley RP, et al. Correlates of organ failure in severe acute pancreatitis. Journal of the pancreas. 2009; 10:271–5. 19454818

[27] Browne G-W, Pitchumoni C-S. Pathophysiology of pulmonary complications of acute pancreatitis. World J Gastroenterol. 2006; 12:7087–96. doi: 10.3748/wjg.v12.i44.7087 . 17131469 PMC4087768

[28] Iyer H, Elhence A, Mittal S, Madan K, Garg PK. Pulmonary complications of acute pancreatitis. Expert Rev Respir Med. 2020; 14:209–17. doi: 10.1080/17476348.2020.1698951 . 31779502

