# Supplementary material for: Pleuropulmonary pathologies in the early phase of acute pancreatitis correlate with disease severity
Source: PLoS One. 2022 Feb 7;17(2):e0263739. doi: 10.1371/journal.pone.0263739 (PMC8820650; doi:10.1371/journal.pone.0263739)
Supplement: S1 Table — (DOCX) [file pone.0263739.s001.docx]

**S1 Table. Overview of study centres and included patients.**

| **Study centre** | **Number of patients (%)** |
| --- | --- |
| Halle, Germany | 90 (25.1) |
| Sofia, Bulgaria | 41 (11.5) |
| Barcelona, Spain | 42 (11.7) |
| Lund, Sweden | 47 (13.1) |
| Tallinn, Estonia | 42 (11.7) |
| Helsinki, Finland | 47 (13.1) |
| Kaunas, Lithuania | 49 (13.7) |
| Total | 358 (100.0) |
