# Supplementary material for: Pleuropulmonary pathologies in the early phase of acute pancreatitis correlate with disease severity
Source: PLoS One. 2022 Feb 7;17(2):e0263739. doi: 10.1371/journal.pone.0263739 (PMC8820650; doi:10.1371/journal.pone.0263739)
Supplement: S2 Table — (DOCX) [file pone.0263739.s002.docx]

**S2 Table. Predictors of severe acute pancreatitis: Univariable analysis.**

|  | **Severity of acute pancreatitis (n = 358)** ^†^ | |  |
| --- | --- | --- | --- |
| **Variable** | **Mild/moderately severe**  (n = 312) | **Severe**  (n = 46) | **p-value** |
| **Gender** (%)  Female  Male | 127 (40.7)  185 (59.3) | 19 (41.3)  27 (58.7) | 1.000 |
| **Age** (%)  ≥60 years  <60 years | 148 (47.4)  164 (52.6) | 25 (54.3)  21 (45.7) | 0.473 |
| **BMI ^1^** (%)  ≥25 kg/m^2^  <25 kg/m^2^ | 191 (74.9)  64 (25.1) | 30 (69.8)  13 (30.2) | 0.601 |
| **Aetiology ^2^** (%)  Alcohol  Other than alcohol | 102 (32.7)  210 (67.3) | 13 (28.3)  33 (71.7) | 0.666 |
| **Aetiology ^2^** (%)  Biliary  Other than biliary | 120 (38.5)  192 (61.5) | 12 (26.1)  34 (73.9) | 0.144 |
| **Comorbidity ^3^** (%)  Known CLD  No CLD | 23 (7.4)  289 (92.6) | 8 (18.2)  36 (81.8) | **0.036** |
| **Pleural effusion**  Yes  None | 160 (51.3)  152 (48.7) | 35 (76.1)  11 (23.9) | **0.003** |
| **Pleural effusion localisation** (%)  Bilateral  None/unilateral | 119 (38.1)  193 (61.9) | 31 (67.4)  15 (32.6) | **<0.001** |
| **Pleural effusion amount** (%)  Severe/moderate  Mild | 78 (25.0)  234 (75.0) | 23 (50.0)  23 (50.0) | **<0.001** |
| **Pleural effusion localisation and amount** (%)  None/mild/unilateral  Severe/moderate/bilateral | 191 (61.2)  121 (38.8) | 13 (28.3)  33 (71.7) | **<0.001** |
| **Dystelectasis** (%)  Bilateral  None/unilateral | 205 (65.7)  107 (34.3) | 42 (91.3)  4 (8.7) | **<0.001** |
| **Pleural enhancement** (%)  Yes  No | 36 (11.5)  276 (88.5) | 8 (17.4)  38 (82.6) | 0.374 |
| **Elevated left diaphragm** (%)  Yes  No | 30 (9.6)  282 (90.4) | 5 (10.9)  Abbreviations: BMI = Body Mass Index, CLD = Chronic Lung Disease (chronic obstructive pulmonary disease, asthma, fibrosis or emphysema)  † According to the revised Atlanta classification  ^1^ Missings: 60 (16.0%)  ^2^ Aetiology: Biliary, alcohol, idiopathic, others (post-ERCP pancreatitis, autoimmune pancreatitis, pancreatitis due to lipid metabolic disorders or medication)  ^3^ Missings: 3 (0.8%)  41 (89.1) | 0.999 |
